# Inferring network properties of cortical neurons with synaptic coupling and parameter dispersion

**DOI:** 10.3389/fncom.2013.00020

**Published:** 2013-03-26

**Authors:** Dipanjan Roy, Viktor Jirsa

**Affiliations:** ^1^Theoretical Neuroscience Group, Faculté de Médecine, Institut de Neurosciences des Systèmes, Inserm UMR1106, Aix-Marseille UniversitéMarseille, France; ^2^Bernstein Center for Computational NeuroscienceBerlin, Germany; ^3^Department of Software Engineering and Theoretical Computer Science, Technische Universität BerlinBerlin, Germany

**Keywords:** multispikes, self-organization, transients, firing rate, parabolic burst, network synchrony, generative model, oscillations

## Abstract

Computational models at different space-time scales allow us to understand the fundamental mechanisms that govern neural processes and relate uniquely these processes to neuroscience data. In this work, we propose a novel neurocomputational unit (a mesoscopic model which tell us about the interaction between local cortical nodes in a large scale neural mass model) of bursters that qualitatively captures the complex dynamics exhibited by a full network of parabolic bursting neurons. We observe that the temporal dynamics and fluctuation of mean synaptic action term exhibits a high degree of correlation with the spike/burst activity of our population. With heterogeneity in the applied drive and mean synaptic coupling derived from fast excitatory synapse approximations we observe long term behavior in our population dynamics such as partial oscillations, incoherence, and synchrony. In order to understand the origin of multistability at the population level as a function of mean synaptic coupling and heterogeneity in the firing rate threshold we employ a simple generative model for parabolic bursting recently proposed by Ghosh et al. (2009). Further, we use here a mean coupling formulated for fast spiking neurons for our analysis of generic model. Stability analysis of this mean field network allow us to identify all the relevant network states found in the detailed biophysical model. We derive here analytically several boundary solutions, a result which holds for any number of spikes per burst. These findings illustrate the role of oscillations occurring at slow time scales (bursts) on the global behavior of the network.

## 1. Introduction

The neuronal spike-burst activity is characterized by recurrent transitions between rest state and firing state where bursts are temporal groupings of multiple spikes. Certain cells in the mammal brain, for example, neurons in the thalamus during periods of drowsiness, attentiveness, and sleep are known to exhibit this type of spike-burst behavior (Sherman and Koch, 1986; Steriade and Llinás, 1988; McCormick and Feeser, 1990; Steriade et al., 1993; Amzica and Steriade, 1998). Autonomously bursting neurons are found in a variety of neural systems, from the mammalian cortex (Morris and Lecar, 1981; Dhamala et al., 2004a,b) to brainstem (Hindmarsh and Rose, 1984; Wang, 1994; Izhikevich, 2007; Jirsa and McIntosh, 2007; Jirsa et al., 2008). When neurons are coupled with each other, they produce different modes of behavior, including synchrony and phase-locking, which have been implicated in memory, cognition, sensory processing, motor planning, and execution (McCormick and Feeser, 1990; Wang, 1994; Jirsa and McIntosh, 2007). Many neurological diseases, on the other hand, including Parkinson, schizophrenia, and epilepsy, are the result of abnormal synchronization (Uhlhaas and Singer, 2006; Jensen et al., 2007), which suggests that a better understanding of the basic mechanisms producing synchrony and phase locking will be a stepping stone toward the repair of brain function. Modeling attempts using large scale networks to understand emergence of cognitive states rely heavily on the approximation of the dynamics as a neural ensemble. The concept of a neural mass like abstraction (Hebb, 1949; Beurle, 1956) designates a group of Co-activated neurons capable of acting like a closed system when performing a certain function. A small scale network of this kind is sometimes referred to as a “neurocomputational unit.” In large scale brain networks, these mesoscopic units of operation serve as the network nodes (see for instance, Deco et al., 2008, 2011; Ghosh et al., 2008). On intermediate spatial scales of few cm, neural activations along the spatially continuous cortical sheets are described by neural fields, for which the connectivity is assumed to be translationally invariant (see, Wilson and Cowan, 1972; Nunez, 1974; Amari, 1977; Jirsa and Haken, 1997; Feng et al., 2006; Jirsa, 2009; Robinson, 2011). To define such small neurocomputational units, simplified neuron models, known as phase models, offer an attractive tool for the study of network modes, since they allow for detailed mathematical analysis of network dynamics (Breakspear et al., 2010). As an example, Carbal et al. have explored the role of local network oscillations in resting-state functional connectivity by using such phase oscillators in the respective nodes of the simulated network. They have shown when these oscillatory units are integrated in the network, they behave as weakly coupled oscillators. Moreover, for a set of network parameters they found subsets of nodes tend to synchronize although the network is not globally synchronized (Cabral et al., 2011). For the present work we use a recently proposed phenomenological model that admits parabolic bursting in one dimension, which is a type of bursting observed in the R-15 neuron in abdominal ganglion of aquatic mollusc Aplysia Californica (Ermentrout and Kopell, 1986; Izhikevich, 2000; Ghosh et al., 2009). This type of bursting can arise even without bistability in the generation of spikes. The investigation carried out in this work with a detailed neuron model capable of displaying spiking and bursting behavior and a minimal model that not only reproduces the mean field amplitude of the original networks but also capture the most important temporal features of its dynamics. The detailed model used here is extensively discussed in Rinzel and Ermentrout (1989). On the other hand, our phase model is a minimal model that captures the generality of the mechanism of bursting present in the detailed model. As we vary network parameters including mean field coupling strength and dispersion, both networks display various temporal dynamics. In order to understand these states in mathematically tractable terms we take advantage of the mean field coupled network of phase model. Our goal is to identify to what degree this mean field model serves as a reliable neurocomputational unit and captures the qualitative features of temporal dynamics of the full network as a function of the investigated network parameters. Mean field analysis for singleton burst reveals solutions such as incoherence and partial oscillation which can be completely described analytically. However, as we are interested in a multispike system where analytical calculation is rather non-trivial and therefore, we combine semi-analytical approach with numerics to derive the stability diagram. Mean field phase network allow us to identify the mechanism of transitions between various network states that appear as solutions of the full network. Stability diagram is independent of number of spikes per burst and qualitatively commensurates well with the findings in our full network. The paper is structured as follows. In the next section, we introduce the Rinzel–Ermentrout model (Rinzel and Ermentrout, 1989) for parabolic bursting and describe the model in details. In the following section, we couple individual neurons via global coupling and present our analysis of this network model. In the subsequent section, we set up a generic network of bursters coupled to their mean field and derive semianalytically all the network states and corresponding phase transition boundaries. In the next section, we derive numerically a stability diagram using global phase coherence measure. In the final section, we summarize the results obtained from mean field descriptions and link them systematically with the network states obtained from biophysical model network.

## 2. Materials and methods

### 2.1. Single neuron model

A dynamical system with multiple time scales (for example, a neuron with spiking-bursting behaviors) can be written in a singularly perturbed form: $\dot{x}=f(x,y),\;\dot{y}=rg(x,y)$, where **x** is the vector of fast variables, **y** the vector of slow variables that modulate the fast activity, and *r* « 1 is a ratio of fast/slow time scales. A system which has been proposed to describe parabolic bursting behavior is known as Rinzel model (1989). Single neuron model parameters used here are exactly as described in Rinzel and Ermentrout (1989).

(1) $$\begin{array}{l} \;\dot{V}=(I-I_{Ca}-(g_{K}w+g_{kca}z)(V-V_{K})-g_{l}(V-V_{l}))/c\\ \;\dot{w}=\phi (w_{\infty }-w)/\tau_{w}\\ \dot{C}a=\epsilon (-\mu I_{Ca}-Ca)\\ \;\dot{n}=\epsilon (n_{\infty }(V)-n)/\tau_{n} \end{array}$$

where *I*_*Ca*_ = (*g*_*Ca*_*m*_∞_(*V*) + *g*_*sCa*_*n*)(*V* − *V*_*Ca*_), $z=\frac{Ca}{Ca+Ca_{0}}$ and gating functions are

(2) $$\begin{array}{c} m_{\infty }(V)=0.5(1+\tan\text{h}((V-v_{1})/v_{2}))\\ w_{\infty }(V)=0.5(1+\tan\text{h}((V-v_{3})/v_{4}))\\ n_{\infty }(V)=0.5(1+\tan\text{h}((V-v_{5})/v_{6}))\\ \tau_{w}(V)=0.5(1+\tan\text{h}((V-v_{3})/2v_{2})) \end{array}$$

where *V* is the membrane potential, *w* is associated with the fast current, *Na*^+^ or *K*^+^, *Ca* and *n* are the two slow currents, Model parameters which are held fixed throughout our simulations are, *V*_K_ = −84, *V*_*l*_ = 60, *V*_*Ca*_ = 120, *g*_*K*_ = 8, *g*_*l*_ = 2, *c* = 20, *v*_1_ = 1.2, *v*_2_ = 18, *v*_6_ = 24, *v*_5_ = 12, *v*_3_ = 12, *v*_4_ = 17.4, τ_*n*_ = 0.05, ϕ = 0.06666666, *gCa* = 4.0, μ = 0.025, *Ca*_0_ = 1, ∈ = 0.0005, and *g*_kCa_ = 1, *g*_sCa_ = 1.

**Figure 1:**
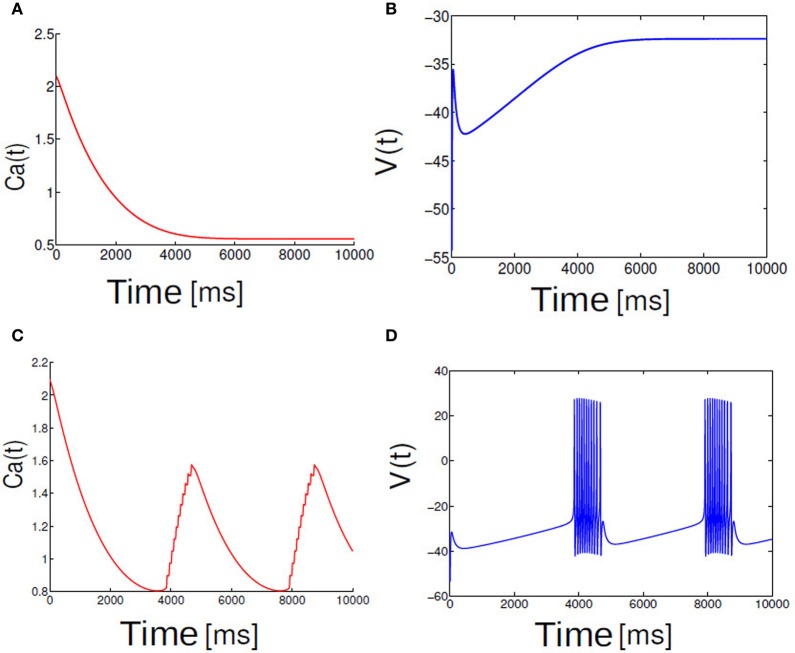
**Shown here trace of membrane potential and calcium dynamics.** Fast spikes rides on a slow modulation of calcium. Slow subsystem moves Ca back and forth across SNIC bifurcation points **(A,C)**. In **(B,D)** membrane potential dynamics is shown for two different cases **(A)** *I* < *I*_*c*_ and **(B)** *I* > *I*_*c*_ in the single neuron model.

*I* is the applied input current. The ionic currents are given by an ohmic leak current, determined by the leak conductance *g*_*l*_ and leak reversal potential *V*_*l*_, and a *Na*^+^ current which is responsible for the generation of spikes. The dynamics of this model which is relevant to our study is outlined as follows. When the input current *I* exceeds a critical value *I*_*c*_ a single neuron described by Equation (1) undergoes a Saddle-node bifurcation on an invariant circle (SNIC). This same system for two different parameterization of *I* and in the presence of the slow currents can exhibit both spiking as well as parabolic bursting behavior. Spiking behaviors are elicited for a slightly higher value of the external drive. For example, to observe a typical burst-like pattern in this system we held the input current to the values *I* = 68 and for spikes *I* ≥ 70. Figure 1 displays the relationship between the applied input current and a parabolic bursting pattern that is observed in the single neuron dynamics.

### 2.2. Phase model

The generality of the underlying mechanism for parabolic bursting is investigated in details by numerous authors (Ermentrout and Kopell, 1986; Baer et al., 1995; Izhikevich, 2000). In many such formulations, parabolic bursting neurons are typically in their canonical form described as:

(3) $$\begin{array}{l} \dot{\theta }=[1-\cos(\theta )+f(x,\;y)]\\ \dot{x}=\mu_{x}[x_{\eta }(\theta )-x]\\ \dot{y}=\mu_{y}[y_{\eta }(\theta )-y] \end{array}$$

where function *f*(*x*, *y*) in the above equation couples to spike generative mechanism depending on the slow variables x, y dynamics, respectively. The function *f*(*x*, *y*) is a smoothly varying periodic function alternating signs such that the system undergoes a SNIC to generate parabolic burst at the single neuron level. Recently Ghosh et al. (2009) has also proposed a simpler model that in principle captures the underlying mechanism of parabolic bursting involving only a circular phase variable θ and moreover, involve only one slow term to allow the fast dynamics to enter or get out of repetitive firing. Motivation for using such a model is primarily mathematical tractability. Parameter space of this model cannot be directly linked to the biophysical parameters, however, qualitatively it may account for the transient and longterm behavior of more detailed biophysical models. In this model a single neuron is described by the following equation,

(4) $$\dot{\theta }=I-\cos\theta -\cos\frac{\theta }{n}$$

In Equation (4) a slow variable activation term is represented by a modulation term $\cos\left(\frac{\theta }{n}\right)$ which mimics the entire slow subsystem instead of describing it as a separate dynamical system, *I* is the applied input current and *n* is an integer, which determines the number of spikes per burst. In our simulation with this model all the results are for *n* = 5 spikes per burst unless otherwise specified.

### 2.3. Full network model

Golomb and Rinzel (1993) considered a heterogeneous network of all-to-all coupled inhibitory bursting neurons and found regimes of synchronous, anti-synchronous and asynchronous behavior when the width of the heterogeneity was changed (Golomb and Rinzel, 1993; Stefanescu and Jirsa, 2008, 2011; Smeal et al., 2010; Jirsa and Stefanescu, 2011). We describe our network equations via a fast instantaneous coupling. N synaptically coupled (all-to-all) parabolic bursting neurons are described by a similar set of non-linear differential equations with fast chemical synapse. To this end we formally describe:

(5) $$\begin{array}{l} \;{\dot{V}}_{i}=(bI_{i}-I_{Ca}-(g_{K}w+g_{kca}z)(V_{i}-V_{k})-g_{l}(V_{i}-V_{l})+KS(V_{i}-V_{\text{th}}))/c\\ \;{\dot{w}}_{i}=\phi (w_{\infty }-w_{i})/\tau_{w}\\ \dot{C}a_{i}=\epsilon (-\mu I_{ca}-Ca_{i})\\ \;{\dot{n}}_{i}=\epsilon \left(n_{\infty }(V)-n_{i}\right)/\tau_{n}\\ \;{\dot{s}}_{i}=a_{s}(V_{i})(1-s_{i})-\frac{s_{i}}{\beta } \end{array}$$

where all the parameters and the gating variables inherit from the single neuron model Equation (1, 2) and *b* is a rescaling factor to applied drive to cross the threshold and elicit spike/burst in the uncoupled system. Stimulus that all the neurons see *I*_*i*_ > 0 are drawn from a uniform distribution assumed to be symmetrically distributed over the interval *I*_*i*_ ∈ [2.1 −Δ*I*, 2.1 + Δ*I*]. Where Δ*I* is the spread of the applied stimulus parameter. Δ*I* introduces a heterogeneity in the spike threshold. The synaptic coupling appears as an ensemble average given by $S=\frac{1}{N}\sum_{i=1}^{N}s_{i}$, where $a_{s_{i}}(V_{i})=\frac{1}{\left(1+\exp(-V_{i}/2)\right)}$ is a sigmoidal activation function. The synaptic strength *K* is the same for all the neurons. For the entire simulation, we fixed the reversal potential of potassium ions to *v*_th_ ≈ 0.0 (for purely excitatory connectivity).

Analysis is carried out for a fast synapse (AMPA-type glutamate receptors), such as those found in the auditory system, the rise time is instantaneous, and post-synaptic responses commence almost instantaneously after the start of presynaptic action potential (Nunez, 1974; Morris and Lecar, 1981). This brisk communication is a consequence of rapid calcium-channel kinetics, which allows significant calcium entry during the upstroke of the presynaptic action potential (Sabatini and Regehr, 1996). Under the fast synapse approximation the variable *s*_*i*_ relaxes much more rapidly than *V*_*i*_, in which case we may apply a quasi-static approximation to (Equation 5) (e), ${\dot{s}}_{i}\approx 0$, allowing us to adiabatically eliminate the synaptic variable via $s_{i}=\frac{\beta }{\left(1+\beta +\exp(-V_{i}/2)\right)}$. The time course of the postsynaptic conductivity caused by an activation of AMPA receptors can be captured by a rise time β_rise_ = 0.09 ms and decay time β_decay_ = 1.5 ms (Gabbiani et al., 1994; Parnas and Parnas, 1994). Numerical results in Figure 3 provides a good approximation for β in the range between [0.01 ms, 0.5 ms]. Although, we have provided here the details about the fast excitatory synaptic connectivity, our approach can be readily extended to inhibitory connectivity as well. In the continuum limit, a mean field formulation with inhibitory synaptic coupling is provided in details in Appendix.

### 2.4. Mean field coupled phase model

Each generic neuron is coupled to this mean field and typically their response to the mean field expressed as *R*(θ) explicitly dependent on θ, and implicitly on time. In absence of any coupling, their vector field flow on a real line is governed by *F*(θ) = ω − cos(θ) − cos(θ)/*n*. In the absence of the term cos(θ)/*n* expression reduces to a mathematical description used in Roy et al. (2011). Together, we can write for *N* (still finite) such neurons:

(6) $${\dot{\theta }}_{i}=F(\theta_{i})-\Gamma R(\theta_{i}),$$

Recently, we have proposed a formulation for mean synaptic activation term under fairly general setting and taking advantage of instantenous activation, deactivation between pre and postsynaptic events. It allows one to describe synaptic activation variable $s_{i}=\frac{\beta }{1+\beta +\exp\left(\frac{-V_{i}}{2}\right)}$ as a non-linear transfer function of membrane voltage (Roy et al., 2011). Moreover, we have described how the mean field coupled spiking neurons can be described mathematically with this synaptic coupling. Details of this formulation is described elsewhere, (Roy et al., 2011). Collective activity of synapses is described by a mean field. For a given population of neurons is expressed more formally as,

(7) $$\Gamma =\frac{K}{N}\sum_{l=1}^{N}\frac{\beta }{\left(1+\beta +\exp\left(-\frac{\cos\theta_{l}}{2}\right)\right)},\;i\neq l.$$

where Γ is the mean field influence function. Coupling *K* is the same for all the neurons. In our previous work, response to such mean field coupling explicitly described as *R*(θ_*i*_) = sinθ_*i*_(cosθ_*i*_ − *v*_th_),

(8) $${\dot{\theta }}_{i}=F(\theta_{i})-\Gamma \sin\theta_{i}(\cos\theta_{i}-v_{\text{th}})+O(\epsilon ),$$

where *O*(∈) contains non-circular deviations of the order ∈ that results due to perturbations. *v*_th_ ≈ 0.0 for all simulations and analytical calculations unless mentioned otherwise. It is important to note that the couplings in the phase descriptions retain their mathematical expression in the full model plus some linearly added correction terms, which scale with the degree of order of deviation from the circle (Roy et al., 2011). Hence, in application it is rather suitable when phase perturbations are close to the circular orbit. The above equation further can be written combining the terms containing a single Fourier harmonic in the coupling plus the higher order Fourier terms.

(9) $${\dot{\theta }}_{i}=\omega_{i}-\sin\theta_{i}-\sin(\theta_{i}/n)+P(\theta_{l})\sin\theta_{i}v_{\text{th}}+O(2\theta_{i})+O(\epsilon ),$$

(10) $$P(\theta_{l})=\frac{K}{N}\sum_{l=1}^{N}\frac{\beta }{\left(1+\beta +\exp\left(-\frac{\cos\theta_{l}}{2}\right)\right)},\;i\neq l.$$

See for details (Roy et al., 2011). Where, in Equation (6) the frequencies *I*_*i*_ ≥ 0 are assumed to be symmetrically distributed over the interval *I*_*i*_ ∈ [*I* − Δ*I*, *I* + Δ*I*] according to a uniform probability distributions.

### 2.5. Characterization of spike/burst coherence in biophysical network model with mean field coupling

The bursting coherence and incoherence is quantitatively characterized in terms of a statistical-mechanical spike-based measure. We consider an excitatory population of neurons coupled to a common mean field drive and heterogeneity in their threshold for spikes/bursts. By varying the strength of the coupling *K* and the stimulus spread Δ*I* we investigate the emergence of spike/burst coherence. Emergence of collective spiking/bursting coherence may be well described by the (population-averaged) global potential,

(11) $$V_{\text{mean}}(t)=\frac{1}{N}\sum_{i=1}^{N}V_{i}$$

In the thermodynamic limit (*N* → ∞), a collective state becomes coherent if $\delta V_{\text{mean}}(t)\equiv [V_{\text{mean}}(t)-\bar{V_{\text{mean}}}(t)]$ is non-stationary (i.e., an oscillating global potential *V*_mean_ appears for a coherent case), where the overbar represents the time average, and also, the correlated mean field Γ(*t*) activity appears oscillatory. Otherwise (i.e., when *V*_mean_ is time independent or stationary), it either becomes incoherent (IN) or partial oscillatory (PO). In *N* → ∞ limit both these states converges to a stationary solution. Thus, the mean square deviation of the global potential is a global marker for mean burst coherence for the entire population described here. More formally one can write it as (i.e., time-averaged fluctuations of *V*_mean_),

(12) $$R(t)={\bar{(}V_{\text{mean}}(t)-\bar{V_{\text{mean}}}(t))}^{2}$$

plays the role of an order parameter used for describing the coherence-incoherence transition (Manrubia et al., 2004). For the coherent (IN) state, the order parameter *R*(*t*) approaches a non-zero (zero) limit value as N goes to the infinity. We compute *R*(*t*) in Equation (12) as a function of mean field coupling strength *K* and dispersion parameter Δ*I* for the full system. We vary both *K*, Δ*I* from 0 to 1 in a step size of 0.01. Subsequently, computed values of *R*(*t*) is plotted in grid size of 100 × 100. Contour plot is colorcoded from low values at zero (blue) to high values at 1 (red). Nearly (in phase or anti phase) synchronized population spike/burst activity is lumped into a regime with labeled as SR and IN population spike/burst activity is lumped into a regime called IN activity. In the IN regime as described above *R*(*t*) values stays close to zero with substantial subthreshold fluctuations. Partial bursty regime is labeled as PO observed for *R*(*t*) values stationary and close to values other than zero. This regime displays dynamical behaviors far from synchrony, such as multi-clustering (some of the neurons are firing incoherently while others are not firing at all) in the phase for instance. Depending on the heterogeneity in stimulus spread we get random distribution of phases such that individual members can exhibit cluster hopping. Multiclustering in our model can reliably be captured using an ensemble average quantity rotation number ρ_*i*_ given by Equation (14).

### 2.6. Characterization of spike/burst coherence in phase network model with mean field coupling

The bursting coherence and incoherence is quantitatively characterized in terms of statistical mechanical order parameter coherence measure. As an alternative to storing and plotting many time series data θ_*i*_(*t*), *i* = 1,…,*N* for all *N* = 1000 variables, we define an order parameter

(13) $$R_{\theta }(t)=\frac{1}{N}\sum_{i=1}^{N}\cos\theta_{i}$$

Equation (13) measures the population dynamics. The advantage of using such a formulation becomes apparent immediately. Let's say our model system has periodic orbit then θ_*i*_(*t*) θ_*i*_(*t* + *T*), where T periodic pacing spikes or bursts (latency). Then in order parameter space one can can detect this state in a straight forward manner as a solution *R*_θ_(*t*) *R*_θ_(*t* + *T*). This result holds for all i, t. In this case, *R*_θ_ dynamics is dominated mostly by the *x* co-ordinate dynamics. Absolute values of mean order parameter mod *R*_θ_ ≤ 1. There is a mathematical relationship of macroscopic global phase measure with macroscopic *V*_mean_(*t*) in Equation (11). The interval between each microscopic spike/burst in an arbitrary *i*th stripe of spike/burst can be determined in a statistical-mechanical way by taking into consideration its contribution to the macroscopic global membrane potential *V*_mean_(*t*). In this interpretation, the time series of the global potential *V*_mean_(*t*) has a local maxima and minima, respectively and strictly bounded between [0,1]. The global cycle in the suprathreshold regime starting from the minimum of *V*_mean_(*t*) which appears first after the transient time is regarded as the first global cycle, which is denoted by *G*_1_. The 2nd global cycle *G*_2_ begins from the next following right minimum of *G*1, and so on. Then, we can introduce an instantaneous global phase measure θ(*t*) of *V*_mean_(*t*) via a linear interpolation in the two successive subregions forming a complete global cycle (Lim and Kim, 2011). A microscopic spike makes the most constructive (inphase) contribution to *V*_mean_ when the corresponding global phase θ_*k*_ for *k*th cycle of spikes/burst is 2*n*π (*n* = 0,1,2,…), while it makes the most destructive (anti-phase) contribution to *V*_mean_(*t*) when θ_*i*_ for an arbitrary ith cycle of burst is 2(*n*1/2)π. By averaging the contributions of all microscopic spikes within a burst in the ith burst stripe to *V*_mean_, we can obtain the following degree of ordering of spikes/bursts. Hence, the contribution of *k*th microscopic burst occurring at the time *t*_*k*_ is ordered by *R*_θ_(*t*_*k*_). If the degree of synchrony is high between the bursts/spikes then *R*_θ_(*t*_*k*_)→ 1. We quantify the average firing frequency to compare the long-term behavior of individual neurons in the population model. We compute the average frequency (also known as the rotation number) of population of neurons using

(14) $$\rho_{i}=\underset{t\to \infty }{\lim}\frac{\theta_{i}}{t},\;i=1,\;\ldots ,\;N.$$

Averaging is carried out over about 1000 neurons starting from random initial conditions after the transient have died out. Collective states of ensemble of *N* = 1000 neurons with spikes per burst *n* = 5 as indicated by their rotation numbers with uniform distribution of frequency *I* in the interval [2.1−Δ*I*, 2.1 + Δ*I*]. Different branch of rotation index indicate different dynamical states of the network as a function of mean field coupling strength *K*, Δ*I*. We carry out a grid search in the 2D parameter space *K*, Δ*I*. Our goal is to obtain a phase transition diagram to understand long-term collective behavior of Equation (8) for large N, as a function of the coupling strength *K* ≥ 0 and the stimulus spread Δ*I* ∈ [0, 1). Global order parameter *R*_θ_(*t*) is computed for different parameterization of *K*, Δ*I* and embedded on a contour plot. Color spectrum is the same as the one used for displaying phase diagram in the full network. The values which are high and close to 1 are indicated by red and the values which are close to zero are indicated by blue.

### 2.7. Clustering analysis in N coupled full and phase network model

We describe firing patterns in large networks (finite N) with excitatory mean field coupling in terms of array diagrams. Array diagrams are obtained by simulating a coupled system consisting of mean field coupled biophysical neurons (*N* = 100) governed by the Equation (5). All the coupling coefficients are the same *K* where *i* = 1,…, *N*. In the arrays the intensity of the voltage variables *V*_1_, …, *V*_*i*_ have been encoded in color spectrum. Two different color spectrums are used for the biophysical network (see Figure 4). In the first color spectrum blue part of the array values implies the quiescent activity of the spikes where the voltage variables have relatively lower values. All the other colors in the spectrum indicates the higher values for the voltage variables, consequently these pixels in the array imply the spike activity. The horizontal line of the array shows the time with increasing epoches of activity. The second color spectrum used here shows burst depiction in the nearly coherent parameter regime. Green colors in the array indicate completely silent neurons. Purple pixels on the green background shows burst activity. On the vertical axis neuron index are aligned and again, on the horizontal axis gives the direction of time. These diagrams were obtained from a phase network by monitoring phases of individual neurons *i* = 1,…, *N* and aligning them on the vertical axis. The choice of the color spectrum used for phases is given by a colorbar with uniformly distributed phase values. In Figure 9 red color index in the spectrum corresponds to higher phase values of θ (close to π) and orange color index are for lower phase values (close to −π). First initial conditions θ_*i*_(0) is generated randomly and then they are sorted according to their neuron index and subsequently distributed uniformly about [−π, π]. The parameters *K*, Δ*I*, for both realizations are chosen from SR, IN regime of the respective phase diagrams.

### 2.8. Numerical procedures and visualization of the system dynamics

Two network models were implemented in Matlab, numerically integrated using second order Runge Kutta routine and Euler–Maruyama (EM) method (Higham, 2001). The simulations were performed with a fixed time step of *dt* = 0.05. The first 200 time points of the simulation are disregarded to set the network to a steady state. Thus, the results within this time were ignored. The membrane potential *V*(*t*), standard deviation of membrane potential std *V*, mean field Γ(*t*), order parameters *R*(*t*), *R*_θ_(*t*) are captured for the entire population. For full network, simulation is carried out for *N* = 100 neurons and for the phase network for *N* = 1000 neurons. Numerical Phase diagrams are obtained using parallel for loops implemented in Matlab. Coupled mean field Phase model represented in Equation (8) can be visualized as a collection of N points rotating around the unit circle, where the estimated phase for each neuron θ_*i*_(*t*) denotes their position on a ring or a circle at time t. This alternate representation of the dynamical system (as N points moving along a circular reference frame, instead of a single point tracing out a trajectory in an N-dimensional phase space) is possible because the system's state space, the N-torus, is equivalent to N copies of the unit circle. It is worth noting that for most other N-dimensional state spaces such a reduced representation is not feasible. In order to distinguish between oscillators with different natural frequencies, we color the dots according to the standard color spectrum: the neurons correspond to the low end of the spectrum (close to -π)(red), neurons at the high end (close to π) (blue), while those in between occupy the middle part of the spectrum (orange/yellow/green). To show how the system evolves from one instant to the next, we plot a series of snapshots of the system at different times (see Figures 11B–D, for example). This allows us to observe the behavior of individual neurons at the same time as we witness the collective evolution of the system toward an attractive state.

## 3. Results

### 3.1. Single neuron burst dynamics

**Figure 2:**
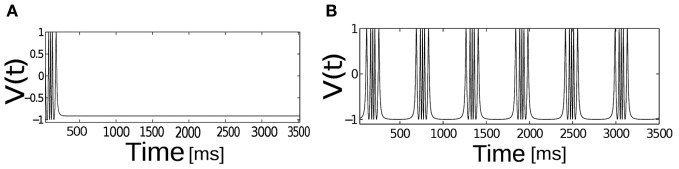
**The temporal dynamics of the phase model of spike-burst neuron.** *V*(*t*) = −cos[θ] is plotted as a function of time, *I*_*c*_ = 2.01, *n* = 5, for **(A)** *I* < *I*_*c*_ and for **(B)** *I* > *I*_*c*_.

We first examine the behavior of single neuron model Equation (2.1) as the applied input current *I* is brought close to the threshold for generating spikes or bursts. For the given parameters In Equation (2.1) a neuron is excitable. Figure 1 depicts the relationship between applied input and parabolic bursting pattern. We are only interested in the behavior of this system for low current values where the resting state of membrane voltage is sufficiently depolarized below −40 mV. When the applied input current *I* is below a critical value membrane potential *V*(*t*) maintains their steady state value and for values greater than the threshold exhibits bursting behavior. For the parameterization used here we find that at *I* ≥ 60 steady state destabilizes exhibiting multispikes. When the applied input current is further increased a neuron make transition from bursting to spiking behavior. In order to observe a typical spiking behavior we set *I* ≥ 70. To get an intuitive understanding about the relationship between slow and fast subsystems, Rinzel and Lee analyzed this model by varying *ca* (a variable in the slow subsystem) as a bifurcation parameter to report that parabolic bursting is obtained from an oscillation in the slow subsystem that periodically moves the *ca* variable back and forth across the SNIC bifurcation, to link the steady state solution of this system to (quiescence state in Figure 1A) the branch of periodic solutions (Figure 1B) and vice versa. Time series of fast variable shows that the interspike interval is relatively longer at the beginning and end of each burst. As has been shown by numerous authors oscillation for the fast dynamics is obtained when the slow variables are held fixed; it is where the saddle-node-loop bifurcation occurs. There is a clear threshold below which there is a unique stable fixed point. Parabolic bursting can occur without having any bistability in the spike generating process. One way to achieve parabolic bursting behavior without requiring any bistability in the generating process and moreover, mathematically tractable would require a generic description like the one shown in Equation (2.2) (see section 2). From numerical results we find that as the applied input current *I* → 2, time period *T* → ∞. Applied input current can be tuned such that it is possible to obtain parabolic bursts of desired interburst gap. The time evolution of a single neuron activity is shown in Figure 2, where a membrane potential like variable *V*(*t*) = −cos[θ(*t*)] is plotted by numerically integrating Equation (2.2). Temporal dynamics shows regular parabolic bursting behavior. For *I* < *I*_*c*_ a neuron fires few spikes before it settles into a steady state. For *I* > *I*_*c*_ (*I* = 2.01, *n* = 5) neurons exhibits parabolic bursting behavior. Based on the qualitative similarity in the burst pattern with parabolic bursting neurons (At the start and the end of the active phase the spike frequency is smaller compared to the middle of the active phase as can be seen in Figure 2 detailed model is substituted to investigate the network effects.

### 3.2. Network dynamics of parabolic bursting neurons with heterogeneity

**Figure 3:**
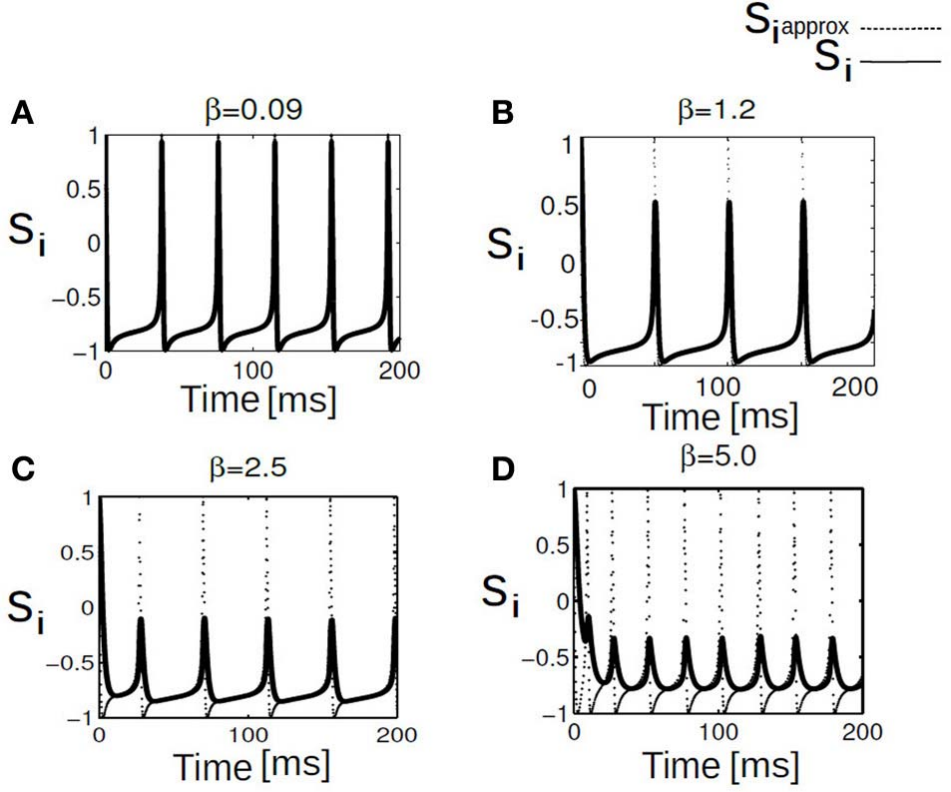
**Shown here trace of synaptic variable and approximated synaptic variable.** Traces are plotted for a spiking regime of our network at *I* = 80, this external current is applied to each neurons in this population. Panels **(A–D)** are generated for low to high β synaptic time constant values. figure shows approximation breaks down progressively as we go to higher β values or access slower time scale. Approximation holds for faster time scale of oscillations.

**Figure 4:**
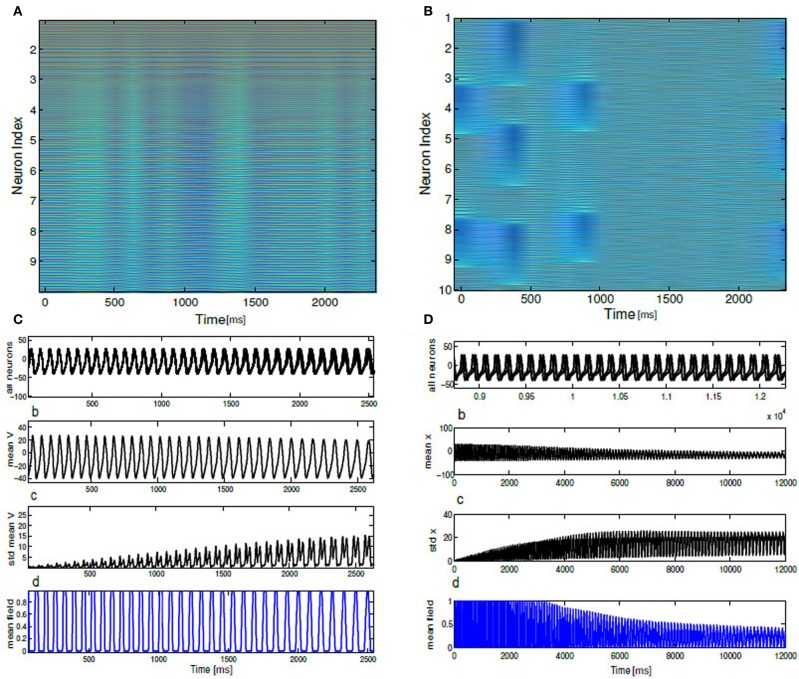
**Array diagrams are shown in (A,B) for two distinct network states.** In a nearly coherent states with clusters of synchronous bursting activity Δ*I* = 0.001, *K* = 0.7, in **(B)** incoherent states for Δ*I* = 0.12, *K* = 0.01. Nearly coherent states showing dynamical clustering effects and wave-like activity spread. Membrane potential time series is shown for all the neurons exhibiting spiking dynamics both in the coherent and incoherent states. Mean membrane potential amplitude decreases and converge to a stationary solution. Standard deviation shown in **(C,D)** shows growth in time. Mean field traces shows periodic activation and deactivation in the coherent state. In the incoherent state mean field amplitude systematically decrease in time.

**Figure 5:**
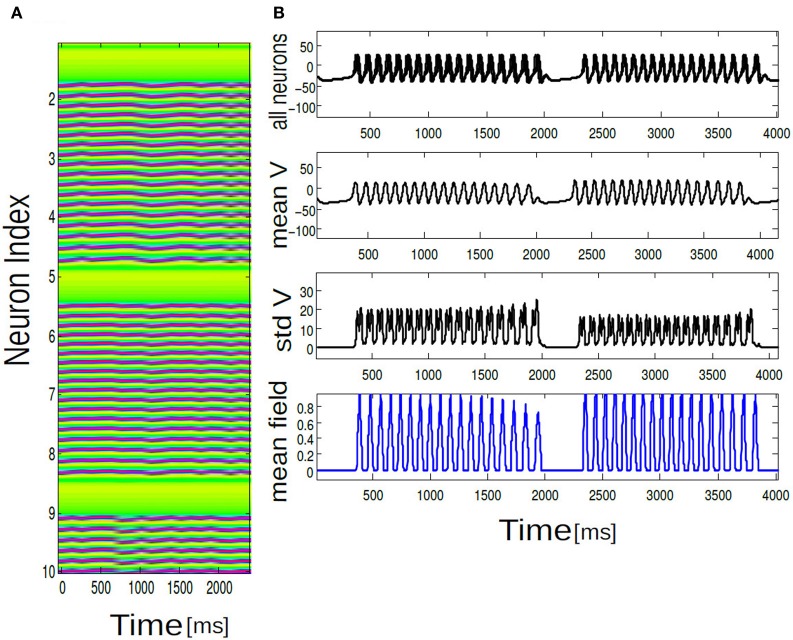
**In **(A)** array diagram showing firing pattern in a population of 100 neurons.** Only 10 neuron index are shown for clarity. Horizontal axis is always time and vertical axis is labeled as neuron index. Green color corresponds to no firing activity or quiescence. Purple pixels corresponds to parabolic bursting activity of each individual neurons which are locked in time. In **(B)** time series data for membrane potential of *V*(*t*), *V*_mean_(*t*), Γ(*t*), and std *V*_mean_(*t*) are plotted for 10 neurons. Mean population burst synchronizes in time.

**Figure 6:**
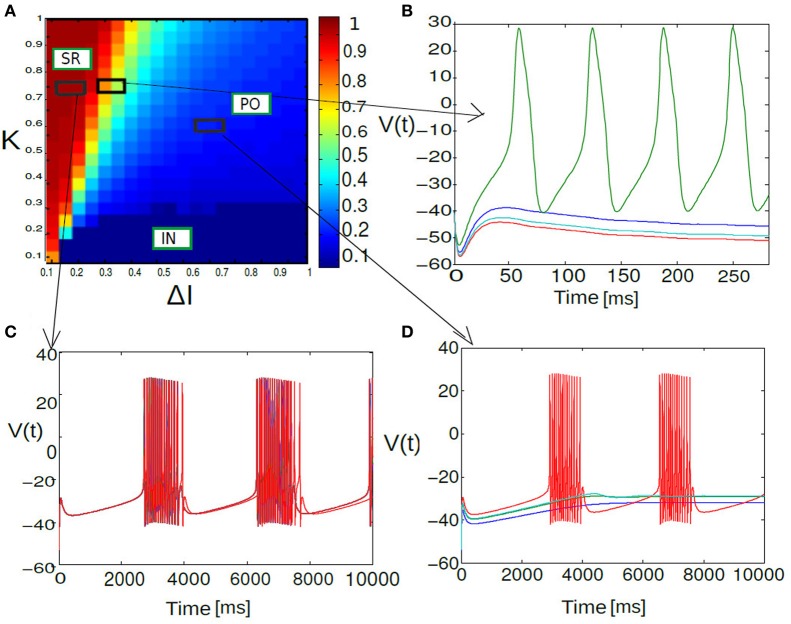
**Phase diagram of mean synaptic action variable is shown as a function of 2D parameter space of stimulus spread Δ*I* and excitatory coupling strength *K*.** In the partial burst regime labeled as PO in **(A)**, a subset of neurons are not firing at all as their respective drives are below their individual firing thresholds. Heterogeneous dynamics between synchronized population spiking activity and oscillation frequency death response for PO state is displayed in **(B)**. Nearly synchronized population of spike/burst activity lumped in a regime labeled as SR [corresponding time series is displayed in **(C)**] and incoherent population spike/burst activity is lumped into a regime called IN. In the incoherent regime mean field values stay close to zero with substantial subthreshold fluctuations. In **(D)** multi stability of PO state is displayed again; now between population burst and fixed point dynamics for an entirely different parameterization.

To understand the influence of heterogeneity in the applied input current and the coupling strength in a network of single neurons exhibiting parabolic bursting we use Equation (5) and parameters as described in section 2.4. We use fast excitatory synapses to couple these units. When the synaptic coupling is sufficiently fast, the coupling tends to push the neurons toward anti-synchrony (Wang et al., 1992; Friesen et al., 1994; Van Vreeswijk et al., 1994). Moreover, several studies have observed emergence of multistable solutions in their mean field network with parameter heterogeneity (Assisi, 2005; Jirsa and Stefanescu, 2011). Our motivation is to go toward this particular direction to capture the relevant network dynamics at the population level. In particular to understand the combined effect of heterogeneity in the firing rate threshold (biophysical model) with the fast time scale of activation-deactivation of synapses in the coupling; the interplay between these two critical factors in spike/burst timing at the population level is largely unknown. In our formalism their individual and combined influence on the network dynamics become clearly visible. Typical time course of such responses of synaptic variable in our model simulation are shown in Figure 2). Fast synapse approximation holds as long as the variable *s*_*i*_ relaxes much more rapidly than *V*_*i*_, in which case we may apply a quasistatic approximation to reduce *s*_*i*_ further in Equation (5), *s*_*i*_ ≃ 0 allowing us to adiabatically eliminate β, and set the synaptic variable via an approximation as $s_{i}=\frac{\beta }{1+\beta +\exp\left(\frac{-V_{i}}{2}\right)}$. The mean synaptic action can be formulated as $\Gamma =\frac{1}{N}\sum_{i=1}^{N}s_{i}$, where $a_{s}(V_{i})=\frac{1}{\left(1+\exp(-V_{i}/2)\right)}$. The synaptic constant *K* is the same for all the neurons. Figures 3A–D shows kinetics of excitatory synaptic variable *s*_*i*_ (plotted with black solid lines) for different β values. Mean synaptic variable (plotted with dotted lines) for the same set of values of time constant β shows dissimilar temporal response compared to *s*_*i*_ for higher time constant values. For smaller time constant values simulation provides relatively better aggrement as can be seen from Figure 3. We numerically integrate the above network to investigate how the mean population burst changes with time as a function of spread of applied stimulus Δ*I* and mean field coupling strength *K*. Firing patterns in this network are shown with array diagrams in Figures 4A,B. For small spread in the applied stimulus and sufficient coupling strength Δ*I* = 0.001, *K* = 0.7 nearly burst synchronization takes place. Moreover, in the array diagram we detect clusters of synchronous states which fires in a wave-like pattern. Corresponding time series of mean quantities such as the membrane potential *V*_mean_(*t*) in Equation (11), mean field Γ(*t*) shows periodic activity in Figure 4C. Membrane potential spiking activity is nearly synchronized across population of neurons in Figure 4. On the other hand, for the IN state mean membrane potential fast decays to zero and shows subthreshold fluctuations about mean zero. Response of mean membrane potential is more suppressed compared to their mean field oscillations between [0,1]. Amplitude of mean field Γ(*t*) changes in time systematically but fluctuates about the mean value of 0.5 instead of approaching zero values as can be seen in Figure 4D. Population burst synchrony is observed for many different parameterization, for one such choice of parameter Δ*I* = 0.002, *K* = 0.8, an array diagram is computed and plotted in Figure 5A. As can be seen in the figure a wave-like spread of activity. In Figure 5B various time series plots of population burst synchrony is shown across 10 neurons. In order to identify different network states for all possible combination of two parameters *K*, Δ*I* we carry out a grid search and compute the values of *R* in Equation (12). Global order parameter measure identifies three distinct network states in the parameter space as shown in Figure 6A. For low coupling values *K*, order parameter shows fluctuations about mean zero. In this regime each neurons activity is mainly driven by their firing rate threshold and displays largely incoherence. For medium values of both coupling strength *K* and stimulus spread Δ*I* network exhibits a hybrid state (some neurons are firing and some of them are silent). For very small values of stimulus spread and medium to high *K* values nearly burst synchrony appears. Temporal dynamics of membrane potential activity *V*(*t*) for four neurons are plotted in Figures 6B–D for three arbitrary parameterization of our network model. In Figures 6B,D PO state is shown where one neuron is spiking or bursting and three neurons are silent. In Figure 6C all neurons are showing nearly synchronized parabolic bursting behavior.

### 3.3. Network dynamics of parabolic bursting phase model with heterogeneity

**Figure 7:**
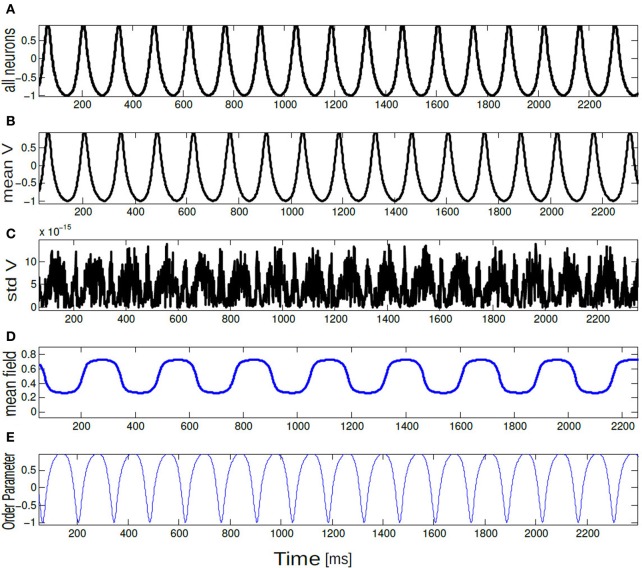
**Time series of (A) *V*(*t*) = −cos θ_*i*_(*t*), (B) *V*_mean_(*t*), (C) std *V*_mean_(*t*), (D) mean field Γ, and (E) order parameter *R*_θ_(*t*) are plotted for 10 neurons.** All neurons are spiking in synchrony and time locked. The parameter values are *K* = 0.8, Δ*I* = 0.001.

**Figure 8:**
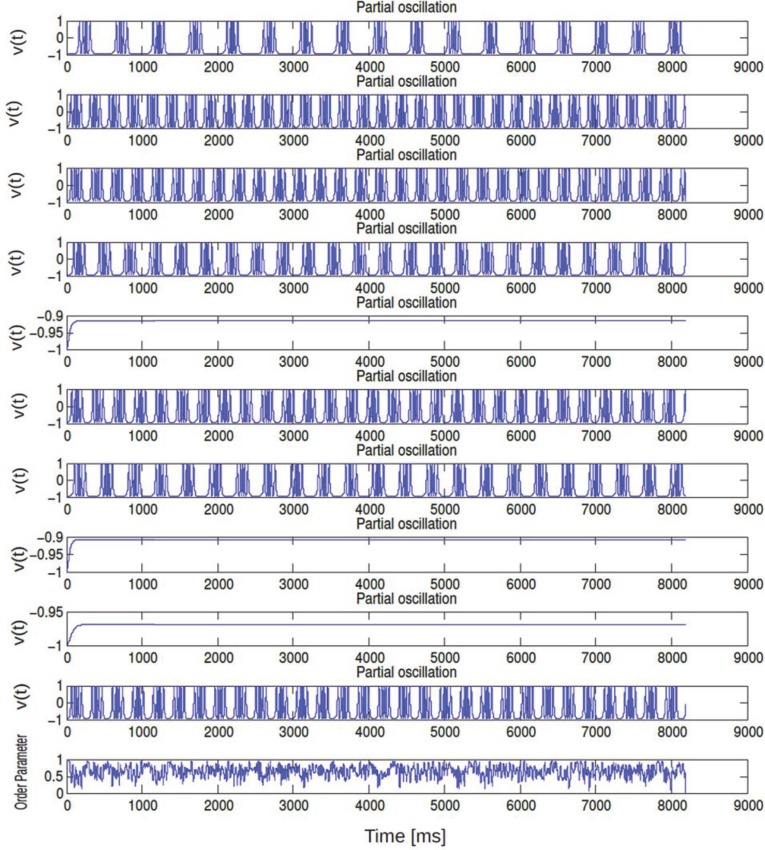
**Temporal evolution *V*_*i*_(*t*) for *N* = 10 neurons are shown here for an arbitrary parameterization *K* = 0.8, Δ*I* = 0.5.** *K* value is unchanged from previous figure. Stimulus spread Δ*I* is changed. Time series of order parameter *R*_θ_(*t*) undergoes statistical fluctuations of magnitude $O\left(\frac{1}{\sqrt{\left(N\right)}}\right)$ about some positive constant value. After 8000 time points, dynamics is truncated assuming network dynamics settles into a steady state.

In this section, we use a phase network with mean field coupling to get some insights about the novel network states observed in (*K*, Δ*I*) the parameter space of the full network model. Coupling between each phase neuron via a mean field is formulated in section 2.4. Numerically we integrate Equation (6) to compute time averaged membrane potential, mean field Γ as in Equation (10) (see section 2), global measure of coherence *R*_θ_ as a function of *K*, Δ*I* a parameter combination which is used in the detailed network model. Time evolution of the above quantiles are shown in Figure 7 for a parameterization *K* = 0.8, Δ*I* = 0.001. The parameter choice is the same as the full network investigations. With this combination of parameters all the neurons synchronously spikes. Mean membrane potential-like quantity *V*_mean_(*t*) oscillates in phase with synchronized spike activity as plotted in Figure 7B. Here, *n* a quantity which determines the number of spikes per burst is kept at *n* = 1. Mean field Γ also shows up and down states (Locked in time) and act as an oscillating drive to each individual neurons. The time series of the global order parameter *R*_θ_(*t*) for synchronized spiking is periodic in Figure 7E. Next, we show in Figure 8 temporal evolution of the mean quantities for the choice of *K* = 0.8, Δ*I* = 0.5. For medium values of mean field coupling strength and stimulus spread network shows PO behavior, where some of the neurons are firing incoherently and others are completely silent. This means for some parameterization network has two stable branches of solutions. It is important to note PO state of the network was observed in the full network for a comparable parameterization (see Figure 6). Time series for 10 neurons and their order parameter evolution in time is plotted in Figure 8. Three neurons are completely silent while other seven neurons are bursting with variable inter-burst intervals. As there is no noise in this system and coupling magnitude is set at high values as in the case of sync, this variability must be introduced by the heterogeneity in their individual firing rate threshold via stimulus spread.

Figure 6 shows the parameter space diagram for the full and phase models presented in Equations (5) and (6–9). Phase boundaries are calculated by computing the mean field for both full and the phase model for different combination of (*K*, *I*) values on a two-dimensional grid. In the following subsection we would lay out the details for obtaining the phase transition boundaries semi analytically. Interestingly, Over a wide range of (*K* −Δ*I*) values the collective dynamics of the two networks primarily show three distinct regions of interest which are close to each other in the parameter space. For sufficiently large K values holding Δ*I* fixed to a narrow range of values near zero, the system converges to a state of partial oscillations in which the some of the neurons are not firing altogether, while the others display IN oscillations. Very large *K* values result in damping of oscillation activity and all the neurons stops firing altogether. The stability state of locking is much more difficult to achieve and in-fact we found distinct branches in their rotation number, these states should all be regarded as variants of 1:1 locking, and therefore we lump them together in the locked region of the stability diagram. With further increase in Δ*I*, parameter heterogeneity, successively more neurons peel away until eventually the entire population is IN.

## 4. Phase diagram using semi analytical methods for mean field phase model

Mean field coupled neurons in phase model is described in Equation (6). Let's rewrite the mean field equation explicitly.

(15) $$\dot{\theta_{i}}=(F(\theta_{i})-\Gamma \sin\theta_{i}(\cos\theta_{i}-V_{\text{th}}))$$

where $\Gamma =\frac{K}{N}\sum_{j=1}^{N}\frac{\beta }{\left(1+\beta +\exp\left(-\frac{\cos\theta_{j}}{2}\right)\right)}$.

In a semianalytical approach we would like to understand the phase transitions between three distinct network states discovered in two networks. For the IN states where the average firing frequency increases monotonically plotted in Figure 10, the θ_*i*_ are all distributed across the closed orbit in a unit circle. This leads to the following phase evolution equation SR state may undergo instability either through parameter changes of *K* or Δ*I* and make phase transition to either IN or PO state. Mean field Γ approaches a stationary density as the number of neurons are increased in both PO and IN state (see Figure 4D). Hence, Γ approaches some positive real number for these two states. When varying *K*, we consider small perturbations μ to the SR solution θ = θ_*i*_ = 0. With θ = θ_*i*_ = 0 + μ Equation (15) becomes $\dot{\theta_{i}}=\dot{\mu }=F(\mu )+\frac{\Gamma }{2}\sin(2\mu )$ and linearization yields $\dot{\theta_{i}}=(F^{\prime }(0)+\Gamma )\mu$. Moreover, SR state may gets phase locked at θ = π (subpopulation clusters). Hence, θ = π may get destabilized as we changed the width of heterogeneity by changing Δ*I* or the coupling strength *K*. Similarly, we consider small perturbations μ about solution θ = π. Hence, we can write θ_*i*_ = π + μ, $\dot{\theta_{i}}=\dot{\mu }=F(\pi +\mu )+\frac{\Gamma }{2}\sin(2\pi +2\mu )$ and linearization yields $\dot{\theta_{i}}=(F^{\prime }(\pi )+\Gamma )\mu$. With $F(\theta )\approx I-\cos\theta -\cos\frac{\theta }{n}$ for the SR state, we find that $F^{\prime }(\theta )\approx -\sin(\theta )-\frac{1}{n}\sin\frac{\theta }{n}$ will be generally small for θ = 0,π. SR state solution hence becomes unstable when *F*′(0,π)+Γ = 0, which suggest almost a vertical critical line between SR and PO, IN state. The bifurcation route from PO (multistable state) to IN solutions as the parameter Δ*I* increases is less conclusive in the framework of the circular approximation, since in the previous stability analysis the only *I*-dependent term is *F*′(0,π), which is very small, hence higher orders of the approximation must be considered. We use the following ansatz: If *r* is the radius of a unit circle, any smooth deformation from a unit circle can be approximated as, *r*(θ_*i*_) = 1 + ∈*h*(θ_*i*_). Hence we can compute the non-linear flow contribution with the above first order correction term as *F*(θ_*i*_) + ∈*H*(θ_*i*_). It is possible to explicitly determine *H*(θ_*i*_) for a certain choice of *h*(θ_*i*_) and moreover, *H*(θ_*i*_) has a periodicity of π, that is *H*(θ + π) = *H*(θ). Thus the linear stability analysis about the fixed point θ^*^_i_ = 0 + μ gives

(16) $$\dot{\mu }=(F^{\prime }(0)+\epsilon H^{\prime }(0)+\Gamma )\mu =(F^{\prime }(\pi )+\epsilon H^{\prime }(\pi )+\Gamma )\mu$$

From the above equation with *F*′(0) ≈ 0 and the π-periodicity of *H*(θ_*i*_), we find that the two fixed points at 0,π lose stability at the same time for increasing Δ*I* and as a result leads directly to the IN state. Since *H*′(θ_*i*_) ~ *I*, scales linearly for fixed μ, we can also estimate the critical line of transition in the parameter space in Figure 11 which separates PO state from IN state. For the critical line: *H*′(θ_*i*_) = *m*(*I* − *I*_*c*_)^*p*^ where *m* is the slope of this line and *m* > 0 allows for destabilization. Hence the critical condition is ∈*H*′(0) + Γ = 0. By substituting the dependence of *H*′(θ_*i*_) on (*I*, *I*_*c*_) and in turn dependence on Δ*I* one can write ∈*m*(*I* − *I*_*c*_)^*p*^ + Γ = 0. This implies coupling strength *K* = −∈(Δ*I* + Δ*I*_*c*_)^*p*^ for (*m* > 0) and *p* is some exponent representing a scaling relationship near saddle-node bifurcation. Thus the critical condition is |*K*| = ∈*m*(Δ*I* − Δ*I*_*c*_), which serves as a convenient guide to numerically compute the stability line separating PO region from IN. Next we try to obtain analytically the stability boundary between INC and PO oscillation states in the infinite-N limit. it turns out that the IN and partial oscillation states can be made steady in our system. The possibility of doing so was suggested by the numerical results. In numerics we observed that as the number of neurons *N* is increased, the order parameter *R*_*t*_ approaches a constant for both these states Figure 8 and the oscillators tend to arrange themselves in a stationary distribution around the circle Figure 11. The way to approach these two states analytically, therefore, is to first write down the appropriate infinite-N analog of our model.

**Figure 9:**
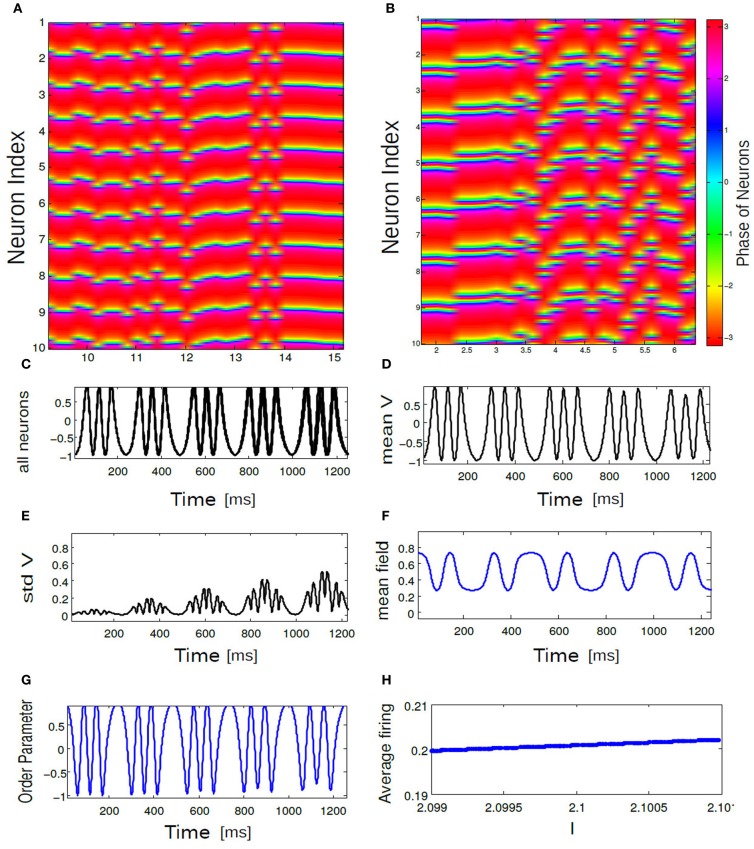
**In (A,B) an array diagram is shown for phase network model for a parameter combination.** In **(A)** *K* = 0.61, Δ*I* = 0.001, *n* = 1 spikes only and in **(B)** *K* = 0.001, Δ*I* = 0.15, *n* = 3 bursts only. Almost always, near synchronous burst states are observed for high K and low Δ*I* values. In **(C)** corresponding time evolution of *V*_*i*_(*t*) is shown for all 10 bursting neurons. In **(D)** temporal response of *V*_mean_ is shown for the coherent state of our network. In **(E)** standard deviations of *V*_mean_ is plotted as a function of time. In **(F)** mean field Γ vs. time for the coherent state is shown. **(G)** displays temporal dynamics of order parameter. Average firing frequency as described in Equation (14) is plotted in **(H)** for the parameter combination of *K* = 0.001, Δ*I* = 0.15. Panel **(H)** further demonstrates phase locking behavior among all the neurons.

**Figure 10:**
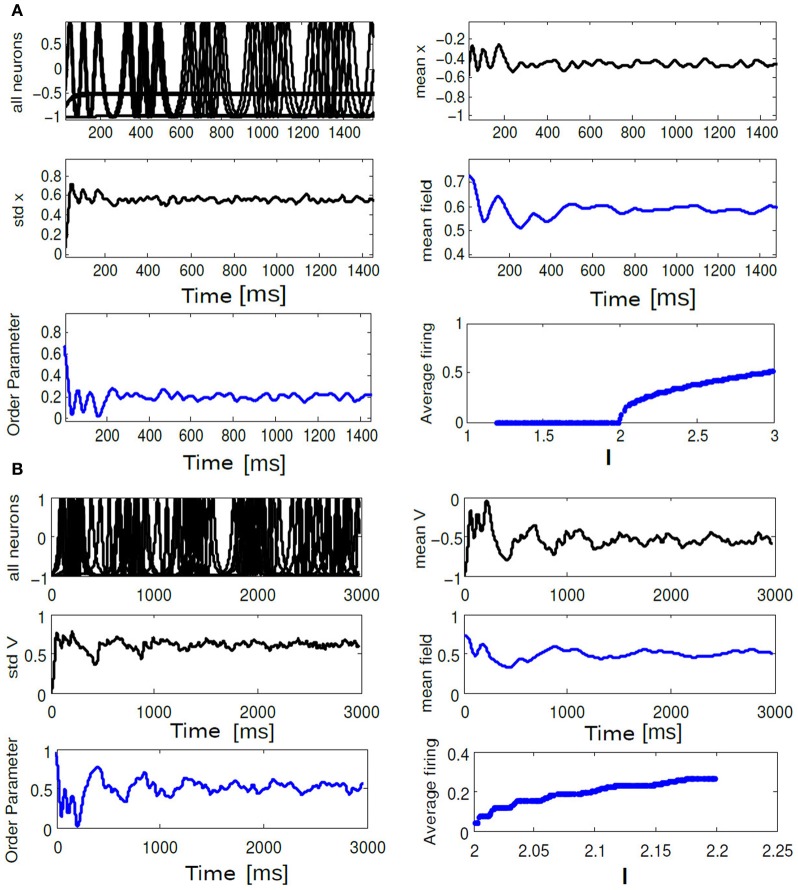
**In (A) time evolution of *V*_*i*_(*t*), std *V*_mean_, order parameter *R*_θ_(*t*), *V*_mean_, mean field Γ, Average frequency are shown for the choice of *K* = 0.6, Δ*I* = 0.3.** In **(B)** time evolution of the same quantities are shown for *K* = 0.01, Δ*I* = 0.1. In **(A)**, average firing frequency plot shows clusters of neurons firing incoherently while another cluster of neurons are completely silent. In **(B)** same subfigure shows a monotonic increase in average firing frequency.

**Figure 11:**
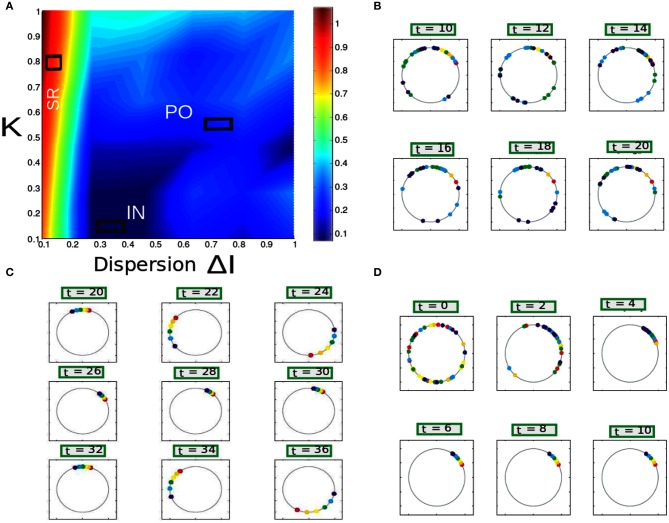
**(A)** Parameter space diagram for *K*, Δ*I* are shown. Color coded computed values of *R*_θ_ shows primarily three distinct network states, labeled as SR, PO, and IN. **(B–D)** For three arbitrary pixel values corresponding firing frequency of individual neurons are shown in a unit circle. Each position in a circle corresponds to a particular phase and color coded according to the scheme described in section 2.8.

(17) $$\frac{\partial }{\partial t}f+\frac{\partial }{\partial \theta }\left\{\left[F(\theta )-\left(\left(\int_{0}^{2\pi }\int_{I-\Delta I}^{I+\Delta I}\Gamma p(\theta^{\prime },\;t,\;I^{\prime })g(I^{\prime })dI^{\prime }d\theta^{\prime }\right)\right)\times \sin(2\theta )\right]p\right\}=0$$

The above equation is the infinite-N analog of continuity equation introduced earlier. It is a non-linear partial integro-differential equation for the number density *f*(θ,*t*,ω). In addition we demand *f* to be non-negative, 2π periodic in θ, and we impose the normalization

(18) $$\int_{0}^{2\pi }f(\theta ,\;t,\;I)d\theta =1,$$

For incoherence and partial oscillation the above system tends toward a stationary distribution of phases in time. The above two states are the fixed points of the stationary density in the continuum limit. To solve for the fixed points we set $\frac{\partial }{\partial t}f=0$ in Equation (10). let's assume that *f*_0_(θ,ω) be the stationary phase density and $v_{0}=[F(\theta )-((\int_{0}^{2\pi }\int_{I-\Delta I}^{I+\Delta I}\Gamma f(\theta^{\prime },t,I^{\prime })g(I^{\prime })dI^{\prime }d\theta^{\prime }))\sin(2\theta )]$ be the velocity field. Then one can write

(19) $$\frac{\partial }{\partial \theta }(f_{0}v_{0})=0\Rightarrow f_{0}v_{0}=L(I)$$

where *L*(*I*) is a constant which is determined exactly by using normalization condition. Depending on it's applied drive *I*, neuron's steady state behavior falls in the following two categories:

Case (i) When *I* << Γ implies
  (20) $$v_{0}(\theta ,\;I)=F(\theta )-\Gamma \sin(2\theta )=0$$
Case (ii) When *I* >> Γ neuron fires incoherently and typically individual phases follows an uniform distribution about the unit circle. In this case the velocity field turns out to be,
  (21) $$v_{0}(\theta ,\;I)=F(\theta )-\Gamma \sin(2\theta )$$

Fixed point solution demands that the density must be inversely proportional to the velocity:

(22) $$f_{0}(\theta ,\;I)=\frac{L(I)}{F(\theta )-\Gamma \sin(2\theta )}$$

In the IN state, neurons driven by different external drives are firing at different phases, however, their collective state is close to being stationary. Every neuron belong to Case (ii) as described above. Further, it is possible to derive nearly an exact relationship between *K*, Δ*I* that gives the transition from case (i) to case (ii) as described above. As shown before, in case of a finite size network such a relationship in the first order perturbation ∈*m*(*I* − *I*_*c*_)^*p*^ + Γ = 0 does exist. In this scenario those neurons with a minimum bound on their applied drive *I*_min_ reach cessation of firing as we find from numerical simulations. They then fall into the Case (i) above where mean field Γ exerts much bigger influence on the dynamics and overall effect is damping of firing activity. The first neurons to stop firing are the ones which do not cross the threshold for firing which in this case *I* > 2. Then the boundary that separates IN from PO in the phase diagram is almost a straight line given by,

(23) $$\left|K\right|=\epsilon m(\Delta I-\Delta I_{c})$$

Hence, both finite and infinite analog of our network identifies the putative transition boundary between IN and PO states. Now from numerical simulations we find Andronov–Hopf (AH) bifurcations leads to the transition from INC to SR solutions in the Figure 6 near *K*, *I* values close to zero. It is equivalent to look at the imaginary eigensolutions that arise due to the instability of the IN state. This instability requires calculation of higher order perturbation terms of the stationary density obtained at the IN state of our network. This is out of the scope of our paper, however, we show a numerical fitting result which gives an empirical relationship between *K* and Δ*I* to quantify the transition boundary between IN and SR states. Assuming ∈ is the perturbation to the IN solution we can express a relationship between *K* and Δ*I* as follows,

(24) $$\left|K\right|=a_{0}\epsilon +a_{1}\epsilon^{2}+a_{2}\epsilon^{3}+O(\epsilon^{4})$$

Equation (24) gives us an empirical relationship between parameters upto fourth order perturbations for the bifurcation of a limit cycle. Optimization of the above equation gives coefficients $a_{0}=\frac{8}{\pi },a_{1}=0,a_{2}=\frac{128}{\pi^{3}}$, respectively. Next, we substitute the amount of dispersion Δ*I* into the perturbative term ∈ to obtain the boundary between IN and SR state. Taken together we can write,

(25) $$\left|K\right|=\frac{8}{\pi }\Delta I+\frac{128}{\pi^{3}}\Delta I^{3}+O(\Delta I^{4})$$

**Figure 12:**
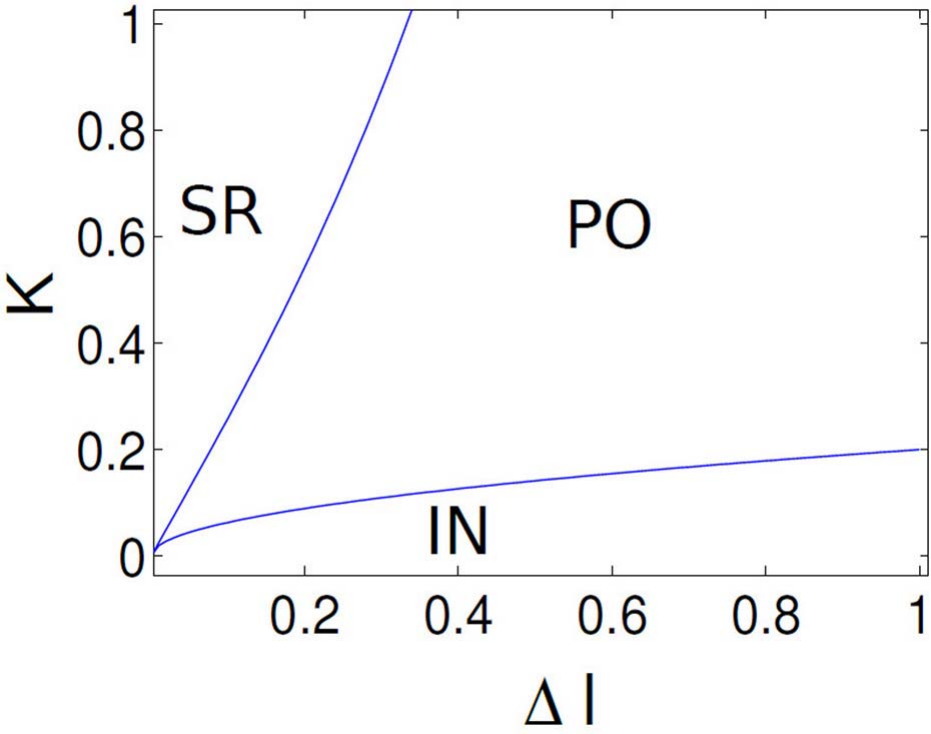
**Parameter boundaries are shown in (*K*, Δ*I*) parameter space using analytic results.** Critical lines separates three distinct network states, labeled as SR, PO, and IN. Critical line separating PO, IN states corresponds to a saddle-node bifurcation and the line that separates IN, SR states corresponds to a Hopf bifurcation.

Results are shown in Figure 12 in the (*K*, Δ*I*) plane using Equations (25) and 23. Critical lines obtained semi analytically qualitatively agrees well with the numerical results that captures various network states in both these models with purely excitatory coupling. In Appendix, we show a stability calculation for an inhibitory coupled mean field network in the infinite analog limit. From numerical simulations we find that the results are independent of the number of spikes *n* per burst.

## 5. Discussion

One of the most frequent assumption employed in simulations of large neural networks is that the whole network can be lumped into small aggregates of collective unit (sometimes called a “neurocomputational unit”) exhibit a sufficiently similar dynamical behavior. Consequently, the network that instantiates this ensemble, consisting of thousands of excitatory and inhibitory neurons, it is considered to display a synchronized behavior with no other significant temporal features for the dynamics of the large scale network. The main reason for this assumption, is the impractical large computational time arising from too many details considered in the large network properties. In this paper, we have analyzed the behavior of a neural network that serves as a good example of such a unit, namely a mean field coupled bursting ensemble. First, we have investigated a Hodgkin–Huxley type detailed biophysical model widely employed in theoretical and computational neuroscience with global coupling. We found that the dynamical features of the network are far more complex then the ones corresponding to synchronized or rest state behavior. The network dynamics depends critical on the balance between firing rate threshold dispersion and mean field synaptic coupling strength; in fact, the synchronized state can be found only for a specific range of parameters typically involving a large or medium values for the coupling strength and low values of dispersion. On the other hand, for large dispersion and weak coupling strength values both networks display purely IN behavior. In the IN state, individual neurons are driven by different external drives results in firing at different phases, however, their collective state is close to being stationary. This stationarity in the density distribution led us to formulate scaling relationship between coupling strength and dispersion parameter. One interesting finding is that, when mean field exerts a greater influence than parameter dispersion; it causes shutting down of the neural activity in some neurons. In this parameter range, we find interesting dynamical behavior such as partial activity. In order to address the problem of the high computational cost of such an implementation, we have further developed a self-consistent mathematically tractable mean field coupled phase model following (Assisi, 2005; Ghosh et al., 2009; Jirsa and Stefanescu, 2011), but incorporating a higher degree of realism. Rather than finding the most appropriate type and number of dimensions that could minimize certain error functions or capture statistical variance in the full network, we have focused our attention on understanding a phenomenological burst generation model system which captures the most important network dynamics of bursting units at the population level. Collective activity of synapses is described by a mean field which relies on instantaneous rise and decay time (Roy et al., 2011). This mean field is then employed in the coupling to individual neurons to describe phase network. Together, we investigate this population of neurons coupled to a common mean field drive and heterogeneity in their threshold for spikes/bursts. Our detailed analysis demonstrated that the reduced representation manages to recreate correctly the topology of the mean field amplitudes of the original system for various parameter scenarios. In the full network, In the thermodynamic limit (*N* → ∞), a collective state becomes coherent if $\delta V_{\text{mean}}(t)\equiv [V_{\text{mean}}(t)-\bar{V_{\text{mean}}}(t)]$ is non-stationary (i.e., an oscillating global potential *V*_mean_ appears for a coherent case) and also, the correlated mean field Γ(*t*) activity appears oscillatory. In the phase network, global order parameter is computed by averaging the contributions of all microscopic spikes within a burst in order to obtain a similar degree of ordering of spikes/bursts as in the full model for identical parameterization. Hence, for a dynamical behavior such as coherence-incoherence transition macroscopic order parameter gives us a crude approximation of burst timing. From a more general perspective, despite its limitations because of the consideration of purely excitatory or inhibitory network, it can be readily extended to study networks with mixed coupling. Moreover, the analytical approach to estimate the scaling relationship and transition boundaries between the IN-PO-SR states is not restricted to small scale network only. With global coupling, as the size of the network grows the boundaries may shift leading to a different parameterization than the one displayed here; however, underlying bifurcations remain the same. We have demonstrated this in our work by analytically deriving a low dimensional mean field amplitude reduction for a inhibitory coupled mean field network in the continuum limit. In this case, all the relevant dynamics of an infinite dimensional network in Equations (29) and (30) is captured by a two dimensional representation of the reduced mean field population given by Equation (40). Thus, using this approach, we derive analytically a low dimensional representation of the network dynamics and we show that the main features of the neural population's collective behavior can be captured well by the dynamics of a few cortical nodes exhibiting spiking as well as bursting behavior. While it is true that strong reductionist assumptions are common (sacrificing many of the biological realism of a network node's dynamics) in large-scale network modeling, these assumptions are usually made *ad-hoc* on the network node's dynamics and limit the network dynamics to a small range. We emphasize here that because of the “near to synchrony” assumption, neural mass models cannot capture complex dynamical features such as multi-clustering, oscillator death or multi-time scale synchronization. Evidently a reduced small scale network model is desirable to serve as a node in a large scale network simulation whereby displaying a sufficiently rich dynamic repertoire. Here it is of less importance to find a quantitatively precise reduced description of a neural population; rather more importantly, we seek a computationally inexpensive population model (this means typically low-dimensional) which is able to display the major qualitative dynamic behaviors (synchronization, rest state, multi-clustering, etc.) for realistic parameter ranges as observed in the total population of neurons. Our approach may offer a viable alternative to the neural mass models currently used in the literature. By comparison, our model offers the possibility to account for such features (temporal details of their spiking activity considered irrelevant for the dynamics of the large network) at a very low computational cost. Therefore, the type of reduced representation discussed in this paper qualifies as a good candidate for a “neural unit” in computational simulations of large scale neural networks.

### Conflict of interest statement

The authors declare that the research was conducted in the absence of any commercial or financial relationships that could be construed as a potential conflict of interest.

## Appendix

### Mean field reduction for inhibitory synaptic coupling

Here, we extend our network in the continuum limit in the presence of inhibitory coupling. *V*_th_ is held negative. (*N* → ∞), where the state of the coupled system can be described by a density function *f*(θ, *I*, *t*), where *f* is defined such that the fraction of neurons with phases lying between θ and *d*θ and applied drive between *I* and *dI* is given by *f*(θ, *I*, *t*) *d*θ*dI* (Antonsen et al., 2008; Ott and Antonsen, 2009). The applied stimulus are drawn from a distribution *g*(*I*) such that

(26) $$\int_{-\infty }^{\infty }\int_{0}^{2\pi }f(\theta ,\;I,\;t)d\theta dI=1$$

(27) $$\int_{0}^{2\pi }f(\theta ,\;I,\;t)d\theta =g(I)$$

For the conservation of currents *I* the continuity equation is written as

(28) $$\frac{\partial f}{\partial t}+\frac{\partial (f\;v)}{\partial \theta }=0.$$

In order to make the coupling amenable to analytical study we use a pulse-like function for the mean field Γ = *a*1 + *b*1(1 + cosθ). Response to the mean field by individual neuron's *R*(θ) = *V*_th_ sin(θ), containing only single Fourier component, a choice motivated primarily due to the tractability of the resulting model. Further, *V*_th_ = −1 for the convenience of calculations without losing any generality of our results. The velocity *v*(θ, *I*, *t*) in Equation (28) is now written as

(29) $$v(\theta ,\;I,\;t)=a+\epsilon \int_{-\infty }^{\infty }\int_{0}^{2\pi }\left(1+\cos(\hat{\theta }))(-\sin\theta )f(\hat{\theta },\;\hat{I},\;t)d\hat{\theta }d\hat{I}-F\sin(\theta )-F\sin(\theta /n\right)$$

where without loss of any generality we are using sin functions instead of cos functions in Equation (6).

In the continuum limit the order parameter *z* can be defined as

(30) $$z(t)=\int_{-\infty }^{\infty }\int_{0}^{2\pi }\frac{\left(e^{i\theta }+e^{-i\theta }\right)}{2}f(\theta ,\;I,\;t)d\theta dI$$

It's a linear sum of two complex order parameters and one could in principle unfold the entire dynamics of the network in any one of the manifold given above. Here,

(31) $$z1(t)=\int_{-\infty }^{\infty }\int_{0}^{2\pi }e^{i\theta }f(\theta ,\;I,\;t)d\theta dI$$

(32) $$z2(t)=\int_{-\infty }^{\infty }\int_{0}^{2\pi }e^{-i\theta }f(\theta ,\;I,\;t)d\theta dI$$

Using the above it is easy to see that the expression for velocity becomes

(33) $$\begin{array}{l} v(\theta ,\;I,\;t)=I+\frac{1}{2i}[\left(1+\frac{\epsilon }{2}(z2+z1^{*})+F\right)e^{-i\theta }\\ \;-\left(1+\frac{\epsilon }{2}(z1+z2^{*})+F\right)e^{i\theta }]\\ \;-F\left(\frac{e^{i\theta /n}}{2i}-\frac{e^{-i\theta /n}}{2i}\right) \end{array}$$

* indicates the complex conjugate. The distribution function can be expressed as a Fourier series

(34) $$f(\theta ,\;I,\;t)=\frac{g(I)}{2\pi }\left[1+\sum_{k=1}^{\infty }f_{k}(I,\;t)e^{ik\theta }+c.c.\right]$$

The above infinite dimensional system is difficult to analyze. However, the “amazing” anstaz of Ott and Antonsen (2009) has been shown to be successful in obtaining the low-dimensional description of the globally coupled phase oscillators. The anstaz impose a restriction on the fourier coefficients:

(35) $$f_{k}(I,\;t)={\left(\psi (I,\;t)\right)}^{k}$$

for *k* ≥ 1 and has been shown to be a reasonable guess under different scenariors (Ott and Antonsen, 2009). This restricted class of functions readily reduces our continuity equation to an θ-independent form

(36) $$\begin{array}{l} \frac{d\psi }{dt}=\frac{1}{2}{\left(1+\frac{\epsilon }{2}z1+F\right)}^{*}-iI\psi \\ \;-\frac{1}{2}\left(1+\frac{\epsilon }{2}z1+F\right)\psi^{2}\\ \;-F\left(\frac{\psi^{1+1/n}}{2}-\frac{\psi^{1-1/n}}{2}\right) \end{array}$$

with *z*1 satisfying

(37) $$z1(t)=\int_{-\infty }^{\infty }\psi^{*}(I,\;t)g(I)dI.$$

If we assume that *g*(*I*) is a Lorentzian distribution function

(38) $$g(I)=\frac{1}{\pi [{\left(I-I_{0}\right)}^{2}+1]}.$$

*z*(*t*) can be evaluated by contour integration with poles at *I* = *I*_0_ − *i* and we obtain the exact evolution equation of order parameter *z*

(39) $$\frac{dz1}{dt}=iI_{0}z1-z1+\frac{1+\frac{\epsilon }{2}z1+F}{2}-\frac{1+\frac{\epsilon }{2}z1^{*}+F}{2}-F\left(\frac{z1^{1+1/n}}{2}-\frac{z1^{1-1/n}}{2}\right)$$

The above equation can be expressed in polar coordinates if we substitute *z*1 = ρ_1_exp(*i*ϕ_1_) giving evolution equations for ρ_1_ and ϕ_1_

(40) $$\frac{d\rho_{1}}{dt}=\frac{\epsilon }{2}\rho_{1}\left(1-\rho_{1}^{2}\right)-\rho_{1}+\frac{F}{2}\left(1-2\rho_{1}^{2}\right)\cos\phi_{1}+\frac{1}{2}\cos\left(\phi_{1}\right)+\frac{F\rho_{1}}{2}\left(\rho_{1}^{-1/n}-\rho_{1}^{1/n}\right)\cos(\phi_{1}/n)$$

(41) $$\frac{d\phi_{1}}{dt}=I_{0}-\frac{F}{2}\left(\rho_{1}+\frac{1}{\rho_{1}}\right)\sin\phi_{1}-\frac{\rho_{1}}{2}\sin(\phi_{1})-\frac{F}{2}\left(\rho_{1}^{1/n}+\frac{1}{\rho_{1}^{1/n}}\right)\sin(\phi_{1}/n).$$

For the Lorentzian distribution function the above equation is exact, However, we do not find any deviation of the above results for any other unimodal distributions of our firing threshold (such as uniform distribution) such as the one considered in the numerical simulations with excitatory coupling. The above two dimensional system can be solved numerically to identify the full network states and the corresponding transition boundaries.
